# *Neisseria meningitidis* Endotoxin and Capsule Transmission by Transplantation

**DOI:** 10.3201/eid1108.050086

**Published:** 2005-08

**Authors:** Nareg Roubinian, Beth D. Kirkpatrick, Freyja Lynn, Jonathan Zenilman, Margaret Bash

**Affiliations:** * University of Vermont College of Medicine, Burlington, Vermont, USA;; † US Food and Drug Administration, Rockville, Maryland, USA;; ‡ Johns Hopkins University School of Medicine, Baltimore, Maryland, USA

**Keywords:** human, infection, case report, endotoxin, transplant, Neisseria, antibodies, capsule

**To the Editor:** Donor organs for transplantation are in extremely short supply. The increased number of patients with advanced liver disease requiring transplantation has resulted in the expansion of selection criteria for potential donors. Previously unacceptable or marginally acceptable donors, such as those who die from bacterial meningitis, are now donor candidates (1).

Several case reports and small, retrospective studies have shown that, with appropriate antimicrobial drug treatment, transplant recipients of organs from donors with bacterial meningitis do not have increased risk for infection (2,3) or reduced graft survival (3). However, endotoxemia in liver transplant recipients has been associated with graft failure and a high mortality rate (4,5), and in gram-negative bacterial infections, the physiologic effects of endotoxin may persist after adequate antimicrobial drug treatment. We report a case of liver transplantation in which the donor had brain death from meningococcal meningitis. This unique case provided an opportunity to study the serologic responses and clinical course of an organ recipient after liver transplantation from a donor who had died of *Neisseria meningitidis* infection. Our observations suggest that biologically relevant levels of antigens, including endotoxin, may have been transferred to the recipient.

A 57-year-old woman with progressive sclerosing cholangitis and cryptogenic cirrhosis received a liver transplant from a previously healthy 18-year-old man who died of serogroup C *N*. *meningitidis* meningitis. He had received antimicrobial drug therapy with ceftriaxone and ampicillin for 5 days before brain death was determined; cultures were negative, and the mildly elevated liver function tests, recorded on admission, had resolved, and no evidence of hepatic impairment was shown.

The transplantation surgery was prolonged (17 h) and technically difficult, requiring intraoperative blood products (≈25 U), prolonged postoperative mechanical ventilation, blood pressure support, and renal hemofiltration. Pathologic examination of the recipient's explanted liver showed secondary biliary cirrhosis. The recipient was given ceftriaxone before and after transplantation for 7 days. During postoperative week 4, she was also treated for *Pseudomonas aeruginosa* nosocomial pneumonia and pleural effusion caused by *Enterococcus*. She was extubated 3 weeks after transplantation and discharged 5 weeks after transplantation. No evidence of clinical infection with *N*. *meningitidis* was identified.

Pathologic examination of the explanted donor liver demonstrated focal acute subcapsular necrosis, large droplet fat accumulation, and mild chronic portal inflammation. The *N*. *meningitidis* isolated from the blood and cerebrospinal fluid of the donor was serogroup C by immunoprecipitation, and the lipooligosaccharide (LOS) immunotypes (determined by Brenda Brandt, Walter Reed Army Institute of Research, Washington, DC.) were L2, L3, L7, and L9.

Banked serum specimens, obtained from the recipient before the operation and every week for 5 weeks after the operation, were assayed for presence of antibodies to *N*. *meningitidis*. Elevated levels of immunoglobulin (Ig) G antibodies (11.2 μg/mL at week 2; 22.5 μg/mL at week 4) (6) to the group C meningococcal polysaccharide capsule were detected in recipient serum (Figure, panel A). Levels of IgM antibodies to LOS L9 rose sharply and peaked in the sample taken 2 weeks after the operation (Figure, panel B). Levels of IgM antibodies to L3 also rose slightly between weeks 2 and 4. IgG antibodies to the 4 LOS types and antibodies to outer membrane vesicles were elevated in the first week postoperation and then declined (data not shown) and may reflect antibodies present from intraoperative blood products. Antipolysaccharide antibodies to *Streptococcus pneumoniae* serotypes 14 and 23 and *N*. *meningitidis* serogroup A also rose between weeks 2 and 4 posttransplantation but were not above values expected in normal adult sera at any time. Bactericidal assays against the infecting strain could not be performed because of endogenous killing that was not complement mediated and was presumed to be caused by the presence of antimicrobial drugs in the serum samples.

**Figure:**
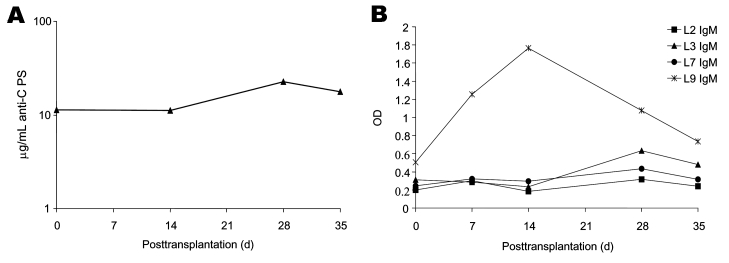
Pre- and posttransplantation serum antibodies as measured by enzyme-linked immunosorbent assay (ELISA). A) Immunoglobulin (Ig) G antibodies to *Neisseria meningitidis* serogroup C capsular polysaccharide (CPS) determined as described by Arakere and Frasch (7) with minor modifications. Samples were run in duplicate at 8 serial dilutions, and antibody concentrations were calculated relative to the standard reference serum lot CDC 1992 (courtesy of G. Carlone, Centers for Disease Control and Prevention, Atlanta, GA). B) IgM antibodies to *N*. *meningitidis* lipooligosaccharide (LOS) immunotypes (L2, L3, L7, L9). ELISA to detect antibodies to LOS immunotypes L2, L3, L7, or L9 was performed as described (8), with minor modifications, by using goat antihuman IgG (γ-specific, Kirkegaard & Perry, Gaithersburg, MD, USA) or goat antihuman IgM (μ-specific, Sigma, St. Louis, MO, USA) conjugated to alkaline phosphatase. Samples were run in duplicate at 4 serial dilutions. OD, optical density.

The rise in IgM antibodies to LOS L9 and IgG antibodies to group C polysaccharide is consistent with a response to exposure to *Neisseria* antigens at the time of transplantation. With effective antimicrobial drug treatment, the recipient has little risk for bacteremia after transplantation of organs from donors dying of *N*. *meningitidis* infection (3). However, bacterial antigens, endotoxin, and cytokines could potentially be sequestered in a donor liver, especially when organ transplantation occurs within days of the bacteremic episode. Despite appropriate antimicrobial drug treatment of the donor and recipient, and the absence of any evidence of active infection of the recipient, these data suggest that proinflammatory endotoxin and capsular polysaccharide from *N*. *meningitidis* were transplanted with the donor liver. Although we cannot definitively associate these findings with the organ recipient's difficult intra- and postoperative course, this case raises the question of the role of proinflammatory responses to transplanted endotoxin in postoperative condition and graft dysfunction in this critically ill population (9,10).

Prospective studies identifying and quantifying endotoxin in the transplanted liver itself and in the recipient may be valuable in assessing the meaning of this finding. An assessment of endotoxin transfer will assist in further defining the risks associated with organ transplantation from donors with *N*. *meningitidis* infections and may lead to the consideration of additional interventions to mediate the effects of endotoxin exposure.
